# On the Causes of Scrofulous Diseases

**Published:** 1841-02

**Authors:** 


*On the Causes of Scrofulous Diseases.*—M. Lugol proposed to himself the question whether scrofulous diseases ought to be regarded as the result of accidental external causes, or that of a hereditary predisposition? And the conclusions to which he has arrived are the following:—

The accidental causes do not necessarily produce scrofula, at least it is very doubtful whether they are alone sufficient to produce the disease. The production of scrofulous diseases, however, by hereditary descent, is the most common and the most clearly proved, and which we are enabled to tracc in the great majority of instances.

The appearance of scrofula in one child is a sure sign of the whole of the rest of the family inheriting the scrofulous constitution, by virtue of which all the other children have the same original predisposition to the disease. If the families which betray the marks of inheriting a scrofulous constitution are observed with care, it cannot fail to be remarked that they are subject to a great mortality. Scarcely a fourth of the children who are born attain the age of puberty, and it is by no means a rare occurrence to find that families, originally very numerous, have been all cut off even before they reached this period of life. Scrofulous disease, in fact, is one of the most active causes of mortality of the human race; there is no other disease which cuts off so many and so young victims.

In his memoir, M. Lugol endeavours to show that parents who are at present apparently free from scorfulous affections, and yet beget scrofulous children, can, in almost every instance, be traced to have had brothers or sisters who have been affected with scrofulous diseases, or have themselves been the subjects of some scrofulous affection in early childhood. There have also been seen instances of scrofula appearing in the parents, after having had born to them scrofulous children. He consequently arrives at the conclusion, that hereditary diseases do not pass over a generation, as has been generally supposed,—*Edinburgh Med. and Surg. Jour.*, July, 1840, from *Comptes Rendus*, January 20, 1840. *Ibid.*

